# Eye, Ear, Nose, and Throat

**Published:** 1895-11

**Authors:** 


### SELECTIONS AND ABSTRACTS.

### EYE, EAR, NOSE, AND THROAT.

Edited by Dunbar Roy, A.B., M.D.

### Septic Irido-choroiditis Consecutive to a Uterine Hemorrhage.

The case was reported by Dr. Valude of Paris.

A woman, about forty years of age, who had had no disease of any sort, whose eyes were perfectly good, while engaged in washing was taken with an abundant metrorrhagia which obliged her to leave her work and quickly take to bed. The physician called believed that he had to do with a miscarriage and prescribed accordingly. The patient remained in bed and the flow continued in diminished quantity during three days. The prostration was great, and there was fever but no signs of uterine inflammation or infection; there was neither pain nor excess of abdominal sensibility, and the discharge was without odor. During the progress of these phenomena, on the evening of the next day, the patient was seized with pain in the left eye, and it was presently seen that the eye became swollen and red.

In a short time the ocular pains acquired a considerable intensity and the swelling of the parts became extreme. There was conjunctival chemosis and exophthalmia. There was an absence of conjunctival secretion, a fact which opposed the hypothesis of an acute conjunctivitis.

At the same time the general condition of the patient took an unfavorable turn, the prostration becoming more extreme and the patient complaining of continued nausea, which was at length succeeded by vomiting. There were no chills. At length the sanguineous flow was arrested and there was no further uterine trouble.

When I saw the patient she stated that she had been in a state of continual fever and had taken no nourishment, and that the attacks of nausea had been frequent; unfortunately, as I did not see her during the early period of the disease, I know nothing of the temperature at that time. Leeches applied to the forehead by the attending physician caused a slight diminution of the severe pains experienced by the patient. A few hours after the onset of the first ocular symptoms, vision was completely abolished in that eye. I was not called to examine the patient until two days after the onset of the trouble. I saw that the eye was slightly prominent but that it was mobile and that the movements were painless, facts which excluded the hypothesis of an inflammatory or rheumatic affection of the orbit, such as phlegmon or tenonitis, which always induces an absolute or relative immobility of the organ. Furthermore, the appearances of the eye suggested an inflammation of the deep membranes; a chemotic swelling of suspicious aspect in the lower part of the conjunctiva; the iris dull, swollen, immovable and projecting slightly forward; the anterior chamber diminished in depth and filled with a suspicious looking fluid; and the pupil with a yellow appearance, which left no doubt as to the condition of the vitreous humor. The latter was opaque and infiltrated with pus, if not transformed to a purulent mass. There was an extreme pericorneal congestion, which explained the violent cyclitic pains experienced by the patient. Further, the ciliary body was extremely sensitive to the touch and the tension of the eye was somewhat increased.

Both the cornea and the conjunctiva were intact, and it was impossible to find the point of entrance of this infection which menaced the integrity of the deep membranes of the eye. The patient had received no injury to the eye—at least no evidence of this could be obtained.

The trouble was, in short, a purulent irido-choroiditis or phlegmon of the eye in the process of development. With the hope of alleviating the symptoms, I prescribed repeated applications of vesicants to the temple, inunctions of belladonna ointment, and laudanum poultices applied to the lids. At the same time tonic treatment was instituted to restore the forces of the patient. Eight days later I saw the patient again in a little more satisfactory condition. The general health was improved and the eye was no longer painful. It was still red, however, but the pericorneal congestion was less marked. The pupil retained its yellowish appearance and the vitreous humor was still opaque. The tension of the eye was rather diminished.

The progress of the acute purulent irido-choroiditis had evidently been arrested, and in spite of the intensity of the initial process it was now certain that purulent destruction of the eye would not result. But the vitreous humor was so infiltrated with leucocytes that it was hardly probable that it would completely recover its transparency. Vision was, therefore, compromised, and the eye subjected to all the vicissitudes which follow an irido-cyclitis of such severe form as this had been. So much for the prognosis.

The most interesting question is the cause of this irido-choroiditis, which evidently was not of direct external origin, as the cornea was perfectly intact. The infection of the deep membranes of the eye is closely connected by relation of cause and effect to the metrorrhagia, possibly arising from a miscarriage. The prompt onset of the ocular disturbances the evening of the day after the hemorrhage and the absence of any uterine infection does not allow of the supposition of a septic inflammation developed in the uterus and transported to the eye by a process of metastasis. I am more inclined to think that the uterine mucous membrane, lacerated by the numerous vascular ruptures brought on by accident, was the door of entrance for an infectious germ, which proceeded to develop in the ocular media, so well adapted to its propagation. The uterus, while serving as the door of entrance, remained free from infection.

Salo Cohn, in Germany, and Gendron, in France, have, in quite recent articles, reported various observations relative to accidents of this kind. My object has been to add a new fact to this category, as the observation of s ıch cases is still rare.—*Annales D'Oculistique*, Febuary, '95.

---

### IS ACUTE TONSILLITIS IN ANY WAY DEPENDENT UPON THE RHEUMATIC DIATHESIS?

Geo. B. Hope, M.D., of New York, read a paper in which he took the ground that the theory of acute tonsillitis, very generally attributed to an underlying rheumatic or gouty diathesis, is, in the writer's experience, not substantiated by clinical observation, and believes that the accepted version is largely due to the natural disposition to fall into line with time-honored views and unconsidered statements. The issue is made that those subject to attacks of tonsillitis do not commonly afford a history of rheumatism, proximate or remote, while, on the other hand, the rheumatic individual rarely suffers from inflammation of the tonsil; that it is noteworthy that the tonsil in later life becomes less and less disposed to acute attacks, while the rheumatic age is more confirmed. Furthermore, as a local acute manifestation, rheumatism should select a sero-fibrinous, rather than a neuro-fibrinous or lymphatic structure like the tonsil.

Carrying the argument further, it is claimed that suppurative peri-tonsillitis is eminently of an infectious nature, and is frequently a sequela of intranasal operation quite independent of climatic or constitutional conditions.

The conclusion reached from above is that the favorite anti-rheumatic remedies, as guiac, and the salicylates, as addressed to the causation, are either erroneous in practice, or act independently and by methods not clearly stated. Such remedies should not be considered specifics, but should be adjusted to the varying conditions of the subject. Moreover, it is not proven that the treatment does abort or mitigate the course of the disease.

---

### IRRIGATION OF THE TYMPANUM (MIDDLE EAR).

Brieger considered the copious tympanic injections beneficial only when all inflammatory manifestations should have subsided. In contrast to the treatment recommended by Bing for the papillary perforations, he advised active dilatation of the orifice, and mentioned that in three cases he had removed the prominence with the snare for the purpose of histological investigation, and had thereby brought about a very good result.

Gruber thought that in chronic exudative inflammations the use of the injections should not be given up. There were cases in which the exudation could be removed from the drums in no other way. Moreover, the irrigations serve to allay the severe pain. In acute cases he did not use them, for fear of driving the exudation into the mastoid cells. In chronic cases the danger is less, as in the majority of cases there is already inflammation in the mastoid cells.

Szenes had seen two good results from the use of injection in acute inflammation of the middle ear, though he thought they should not be used as a rule.

Pins had good results from the use of irrigation in chronic and sub-acute cases, except in one case where cerebral complications, and probably abscess of the brain, occurred. Vertigo and death followed one day after the second irrigation. The method is of great use if the perforation of the drum is sufficiently large to allow free exit for the fluid.

Gomperz replied to Politzer that when in pathological cases the cell spaces of the mastoid processes are filled with pus, the latter was exposed to a higher pressure during injection, which was a matter of some importance.—*Annals of Ophthalmology and Otology.*

